# Anthropogenic microparticles in the emerald rockcod *Trematomus bernacchii* (Nototheniidae) from the Antarctic

**DOI:** 10.1038/s41598-022-21670-x

**Published:** 2022-10-14

**Authors:** Teresa Bottari, Valeria Conti Nibali, Caterina Branca, Marco Grotti, Serena Savoca, Teresa Romeo, Nunziacarla Spanò, Maurizio Azzaro, Silvestro Greco, Giovanna D’Angelo, Monique Mancuso

**Affiliations:** 1 Institute for Marine Biological Resources and Biotechnology (IRBIM) – CNR, Messina, Italy; 2 Department of Integrative Marine Ecology (EMI), Stazione Zoologica Anton Dohrn - National Institute of Biology, Ecology and Marine Biotechnology, Sicily Marine Centre, Messina, Italy; 3 Department of Mathematical and Computational Sciences, Physical Science and Earth Science, University of Messina, Messina, Italy; 4 Department of Chemistry and Industrial Chemistry (DCCI), University of Genoa, Genoa, Italy; 5 Department of Biomedical, Dental, and Morphological and Functional Imaging, University of Messina, Messina, Italy; 6 Institute for Environmental Protection and Research (ISPRA), Milazzo, ME Italy; 7 Institute of Polar Sciences (ISP) – CNR, Messina, Italy; 8 Research Infrastructures for Marine Biological Resources Department (RIMAR), Stazione Zoologica Anton Dohrn, National Institute of Biology, Ecology and Marine Biotechnology, Calabrian Researches Centre and Marine Advanced Infrastructures (CRIMAC), Amendolara, CS Italy; 9 Institute for Chemical-Physical Processes, National Research Council of Italy (IPCF-CNR), Messina, Italy

**Keywords:** Ecology, Environmental sciences

## Abstract

Anthropogenic microparticles (AMs) were found for the first time in specimens of *Trematomus bernacchii* collected in 1998 in the Ross Sea (Antarctica) and stored in the Antarctic Environmental Specimen Bank. Most of the identified AMs were fibers of natural and synthetic origin. The natural AMs were cellulosic, the synthetic ones were polyester, polypropylene, polypropylene/polyester, and cellulose acetate. The presence of dyes in the natural AMs indicates their anthropogenic origin. Five industrial dyes were identified by Raman spectroscopy with Indigo occurring in most of them (55%). Our research not only adds further data to the ongoing knowledge of pollution levels in the Antarctic ecosystem, it provides an interesting snapshot of the past, highlighting that microplastics and anthropogenic fiber pollution had already entered the Antarctic marine food web at the end of the ‘90 s. These findings therefore establish the foundations for understand the changes in marine litter pollution over time.

## Introduction

In the last half-century, one of the most ubiquitous and long-lasting change on the surface of our planet's oceans is the accumulation and fragmentation of plastic^1^. Microplastics (MPs) are plastic particles smaller than 5 mm, manufactured as small particles or originated from the fragmentation of larger plastic items^2^. MPs pollution is widespread all over the world and across global ocean ecosystems, from the tropics to the poles, including the Southern Ocean^3,4^. Despite their ubiquitous presence, there are relatively few reports of MPs in polar regions and particularly in the Southern Ocean^5^.

The Antarctic continent and the surrounding waters have been affected by human activity for approximately two centuries^6^. In most parts of the continent the effects of the scientific activities, fishing and tourism resulted in different types of pollution, including plastic pollution^7^. Moreover, terrestrial and marine habitats adjacent to current or abandoned Antarctic scientific bases are affected by localized contamination^8^.

The oldest reports on the plastic litter occurrence in Antarctic waters and birds date back to the 80s^9,10^. The first records of MP ingestion by seabirds were from the Southern Ocean, when prions *Pachyptila* spp. were found to contain plastic in 1960^11^. Subsequent studies carried out after twenty years on sea ice (2009) have highlighted the presence of 14 different polymers, mainly polyethylene (PE), polypropylene (PP), and polyamide (PA)^12^ (Table 1). Some studies, carried out between 2010 and 2017, reported the presence of plastic polymers also in seawater and sediments, as summarized in Table 1.

**Table 1:** Summary of anthropogenic microparticles in Antarctic seawater and sediment (below the sixtieth parallel).

Area	Sample type	Mean	Measure unit	Main polymers	References
Ross Sea	sea water	0.17	items·m^-3^	PE, PP, PES, PTFE, PMMA, PA	Cincinelli et al.^39^
Southern Ocean	sea water	7.25	items·m^-3^	PE, PP	Isobe et al. (2017)
Ross Sea	sediment	na (5–1705)	N/m^2^	PE, PP, Nylon, SBS, PVC, PS, TPU, PVA, EPR	Munari et al.^38^
Adelaide Island	sediment	0.52 (1–10)	items·m^-3^	Rayon (semi-synthetic fibre)	Reed et al. (2018)
King George Island	sea water	2.4	items·100 m^-3^	PEG, PU, PET, PA	Absher et al. (2019)
Antartic Peninsula	surface sea water	1794	items·km^-2^	PU, PA, PE, PS, PP	Lacerda et al. (2019)
	sea water	353	items·km^-2^	PVC, PP, PS, PVC, nylon	Suaria et al.^5^
East-Antarctic	sea ice	96	items·L^-1^	PE, PP, PA (nylon), varnish (PU and polyacrylates), EVA, resins, NBR, PS, PVA, rayon, PL, silicone	Kelly et al.^12^
Antarctic Peninsula	sediment	1.3	items/g	PEst, PP, PS, PU, PVC, TPE, AP	Cunningham et al. (2020)

Polyethylene: PE; polypropylene: PP; polystyrene: PS; polyvinyl chloride: PVC; nitrile rubber: NBR; poly-methyl methacrylate: PMMA; poly vinyl alcohol: PVA; ethylene vinyl acetate: EVA; polyethylene glycol: PEG; polyurethane: PU; polyethylene terephthalate: PET; polyamide: PA; polyester: PES; polytetrafluoroethylene: PTFE; rubber: TPE; acrylic polymer: AP; Nylon 6.6: Nylon; polystyrene-butadiene-styrene: SBS; thermoplastic polyurethane: TPU; ethylene-propylene rubber: EPR.

Recent studies showed that Antarctic krill can carry out a biological fragmentation of polystyrene (PS) microspheres into nanoplastics (NPs, < 1 µm)^13,14^ and this may be related to the spreading of MPs and NPs in marine ecosystems and the potential repercussion on Antarctic food chains^15^.

Bivalves and gastropods displayed the highest MPs contamination among the Antarctic benthic invertebrates, comparable to the values reported for other less remote areas^16^. MPs ingestion was also highlighted in pelagic amphipods living in Antarctic surface waters^17^ (Table 2).

**Table 2:** Summary of anthropogenic microparticles concentrations in Antarctic vertebrates (below the sixtieth parallel).

Location	Area	Sample type		Species	Items/specimen	Polymer	Reference
Antarctic continent	Arderia Island	Stomach	Southern fulmar	*Fulmarus glacialoides*	1.8	na	Van Franeker et al.^10^
			Cape petrel	*Daption capense*	5		
			Snow petrel	*Pagodroma nivea*	1		
			Antarctic petrel	*Thalassoica antarctica*	0.4		
Scotia Sea	Signy Island	Scat	Gentoo penguin	*Pygoscelis papua*	0.23	PES, rayon, PP, PE, polyacrylonitrile, polyacrylate	Bessa et al.^18^
Antarctic Peninsula and Scotia Sea region	Yalour Island, Deception Island, Hannah Point, Rongé Island	Scat	Adélie penguin	*Pygoscelis adeliae*	0.15	PE, PES, cellulose	Fragão et al.^20^
	King George Island, Paradise Bay B, Hannah Point, Rongè Island, Cierva Cove	Scat	Chinstrap penguin	*Pygoscelis antarcticus*	0.31		
	King George Island—Byers peninsula	Scat	Gentoo penguin	*Pygoscelis papua*	0.29		

Polyester: PES, polypropylene: PP; polyethylene: PE.

Recent articles reported the MPs presence (PE, PP, PA, polytetrafluoroethylene (PFTE), polyacrylonitrile (PAN), and nylon), in scats of various penguin species, including *Pygoscelis papua, P. adeliae, P. antarcticus,* and *Aptenodytes patagonicus*^18–20^.

The area south of 60 S latitude is governed by the Antarctic Treaty System (ATS) and, for the environmental issues, by the Protocol on Environmental Protection to the Antarctic Treaty, which entered into force in 1991. This protocol contains specific annexes on Waste Disposal and Waste Management (Annex III), and on the Prevention of Marine Pollution (Annex IV). Moreover, the Antarctic area is considered as a “Special Area”, and under the IMO-MARPOL Convention for the Prevention of Pollution from Ships^21^; according to the Annex V, the deliberate release of plastic wastes from ships (such as plastic ropes, fishing nets and plastic bags) and other waste is forbidden. Due to the increasing evidence of plastic pollution in Antarctica, the Scientific Committee on Antarctic Research (SCAR) recently designed an action group on plastic pollution in the Southern Ocean^22^, and, in 2019, the Antarctic Treaty System adopted the resolution “Reducing Plastic Pollution in Antarctica and the Southern Ocean”. This document recommends to eliminate plastics personal care products, to identify and exchange information to reduce MPs release from wastewater systems, to support the plastic pollution monitoring in Antarctica and, finally, to insert the MPs issue in the Annexes III and IV to the Protocol on Environmental Protection to the Antarctic Treaty.

Despite the significant efforts to monitor and assess the levels of plastic pollution around Antarctica, the extent, quantity and impacts of MPs in the marine environment of this special area remain largely unknown. Studies on the state of the marine pollution in the Antarctic continent at the dawn of this phenomenon are scarce and it would thus be crucial to shed light on its origin and changes over the decades. Very recently, a study on Antarctic snow revealed the presence of microplastics pollution linked to the work of researchers at the Antarctic Mc Murdo Station^23^.

The emerald rockcod *Trematomus bernacchii* (Boulenger, 1902), also known as the emerald notothen, is a marine fish species belonging to Nototheniidae family, and it is a very common teleost in the shallow waters of the High-Antarctic Zone^24^. It is native to the Southern Ocean where it is a commercially important species^25^. *T. bernacchii* lives in very shallow waters down to 700 m depth and it is adapted to living at extremely low temperature. It is a generalized feeder with a wide niche breadth composed almost exclusively of benthic organisms (mainly infaunal and epifaunal polychaetes, amphipods, and molluscs) and small fishes. *T. bernacchii* may be considered an opportunist feeder as it changes its usual feeding habits to exploit different food resources that are seasonally abundant (zooplankton)^26^. Notothenioids, as *T. bernacchii*, play a key role in the high-Antarctic food web of the Ross Sea representing the link between upper and lower trophic levels contributing to the dynamics and stability of the marine system^27^_**.**_

In this work, the presence and composition of anthropogenic microparticles (hereafter AMs) in the emerald rockcod were investigated, with the aim to evaluate if this kind of pollution already existed many decades ago. This was possible because specimens of *T. bernacchii* have been systematically collected since the late 90 s in the context of the Antarctic Environmental Specimen Bank^28^ (BCAA) and the Italian National Antarctic Museum (MNA) activities, and then properly stored to preserve their integrity. So, specimens of *T. bernacchii* collected from the Antarctic coastal environment of Terra Nova Bay (Ross Sea, Antarctica) in 1998 (the oldest available samples stored in the BCAA) provided an interesting snapshot of the MPs pollution in that time. In this study, the micro-Raman spectroscopy has been used to identify the chemical composition of the microparticles since it is among the most powerful techniques to detect and identify marine microparticles and associated chemical anthropogenic additives, as well as to differentiate between natural and synthetic particles^29–31^_**.**_

## Results

### AMs abundance

The study involved eight adult specimens of *T. bernacchii*; the length ranged from 215 to 275 mm, and the weight from 165 to 319 g (Table 3). Six specimens were positive for AMs (75%), a total of 37 particles (size range 0.4–4.2 mm) were detected in the GITs, mainly belonging to large AMs (63%; size range 1.1–4.2 mm) and small AMs (37%; size range 0.4–0.9 mm).

**Table 3:** Total length (mm) and total weight (g) ranges of the analysed *Trematomus bernacchii* specimens.

Sample	Code	Site	Depth (m)	Length (mm)	Weight (g)	Sex
1	MA/13S/SS5860/09/B806_TB-5	B806	165	275	286	F
2	MA/13S/SS5860/09/B806_TB-6	B806	165	227	165	M
3	MA/13S/SS5860/09/B806_TB-1	B806	165	270	319	F
4	MA/13S/SS5860/09/B806_TB-3	B806	165	215	191	F
5	MA/13S/SS5860/09/B806_TB-2	B806	165	255	219	F
6	MA/13S/SS5860/09/B5_TB-3	B5	10	226	163	–
7	MA/13S/SS5860/09/B806_TB-8	B806	165	237	168	F
8	MA/13S/SS5860/09/B806_TB-4	B806	165	232	195	M

**Figure 1:**
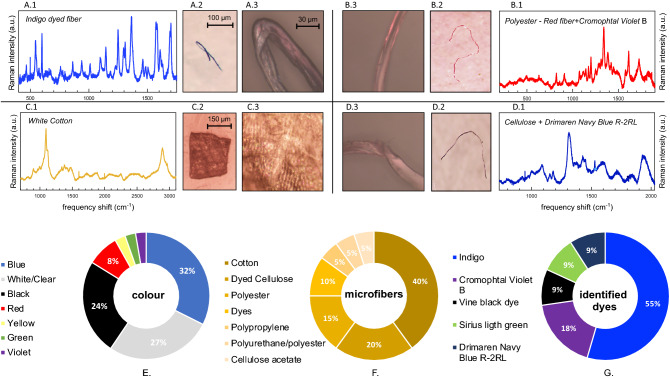
Selected set of anthropogenic microparticles (AMs) isolated from the gastrointestinal tract (GIT) of *Trematomus bernacchii* samples caught in 1998 in Antarctica (Terra Nova Bay, Ross Sea) and provided by the Antarctic Environmental Specimen Bank. AMs have been identified analyzing the spectra measured by means of micro-Raman spectroscopy (panels **A.1**, **B.1**, **C.1**, **D.1**), after image acquisition with an optical stereomicroscope (4x, Leica M205C; panels **A.2**, **B.2**, **C.2**, **D.2**, scale ratio for panel **B.2** and **D.2** as in panel **A.2**) and a Raman confocal microscopy (50$\times$, Olympus BX41; panels (**A.3**, **B.3**, **C.3**, **D.3**), scale ratio for all panels indicated in panel (**A.3**). The 50 × magnification allows us to appreciate: the rigid-rod fiber of polyester (**B.3**); the texture of the cotton fabric (**C.3**); the flat and slightly twisted ribbons of cellulosic fibers (**D.3**). Bottom panels: Distribution percentage of colour (%) (panel **E**), composition (panel **F**) and dye type (panel **G**), for all the identified AMs ingested by *Trematomus bernacchii*.

Of them, 35 (95%) were fibers and 2 (5%) were fragments. No plastic pellets, foams or spheres were found. Blue particles were the most abundant (32%), followed by white/transparent (27%) black (24%), red (8%), green (3%), violet (3%), and yellow (3%) (Fig. 1).

The identified microparticles (n = 20; 57%) were of natural and synthetic origin, on the base of identified AMs, the abundance value was equal to 3.3 items/specimen; while fifteen microparticles were not identified because fluorescence overshadowed their Raman signal.

The finding of AMs in the GITs of *T. bernacchii* may be related to its feeding habits and its strong relationship with the seafloor as also suggested for other benthic fish^32–34^. Several studies in the Mediterranean Sea have found non-plastic fibres, mainly cellulose-based, in different benthivorous species^35,36^. AMs may be accidentally ingested during feeding activity or by secondary ingestion. *T. bernacchii* is a generalized predator^37^ with a wide array of dietary items^26^ and it can be also considered as opportunistic feeder, undertaking occasional vertical migrations to forage on locally and seasonally abundant planktonic prey^26^. In the light of this, *T. bernacchii* may also ingest the AMs along the water column.

Since this study is the first investigation carried out on Antarctic fish species, we could only make comparisons with recent studies on seawater, sediment and benthic organisms from the same study area.

Microfibers were the most frequent microparticles in sediments close to the Italian “Mario Zucchelli” Station (MZS)^38^ and in the waters close to the sewage treatment plant of MZS^39^, as well as in penguins of other Antarctic areas^18,20^.

Fish can act as an indirect source of microfiber contamination for marine predators as for seals^40^ and penguins of sub-Antarctic areas^19^ (21.9 items/g of scat).

### Natural and semisynthetic microparticles

The identified microparticles of natural origin were cellulosic (n = 14; 70%). One of the fragments showed the typical texture of the fabric (see Fig. 1, panel C.3). Cotton was the most abundant item (45%) followed by cellulose (18%). Mostly cellulosic microfibers have been found to be dyed, thus confirming their manufactured (anthropogenic) origin.

Among all microfibers examined, the industrial dye was identified by Raman spectroscopy in 11 items: Indigo, Cromophtal Violet B, Drimaren Navy Bue R-2RL, Vine black dye, and Sirius light green, with Indigo occurring in most of them (55%). The Raman spectra of selected identified items are shown in Fig. 1.

In accordance with our results, most of fibres (~ 88%) found in King Penguins scats of the South Georgia were made of natural cellulosic materials (cotton, linen), with only a few synthetic fibres^19^ (PES, PP, and acrylic).

Textiles are the main microfibers environmental source^41,42^. The synthetic textiles are responsible for the discharge of about 0.5 million tonnes of MPs into the sea each year^43^.

Man-made cellulose fibers associated with dyes or additives could be potentially harmful to marine organism^35,44,45^, in fact the industrial dyes are used in the industry with several polymers such as cellulose fibers. In particular, the indigo is used to dye cellulosic fibers of blue jeans the world’s most popular garment^46^. The presence of Indigo-dyed microfibers has been recently, documented in the marine ecosystems^47^. The “blue jeans” microfibers has been proposed as an indicator of the widespread burden of anthropogenic pollution from temperate to Arctic regions^48^. Moreover, the textile dyes, are very toxic and are related to the environmental degradation^49^.

The textile dyes may compromise the aestethic quality of water bodies, increase biochemical and chemical oxygen demand (BOD and COD), alter photosynthesis, and reduce plant growth^43^. Textile dyes may enter the food chain^43^, bioaccumulate, biomagnificate^50^, promote toxicity, mutagenicity, and carcinogenicity ^51–54^.

In their very recent review on 2022^55^ Athey and Erdle suggested that the abundance of natural and semisynthetic microfibers in the marine biota may be underestimated^55^. They enumerate different possible causes for this under-evaluation, the methods used to isolate microfibers are designed for the recovery and identification of synthetic materials. Some chemicals, used to isolate synthetic microfibers, may cause the degradation of non-synthetic fibers; several studies exclude natural and semisynthetic microparticles from their analyses^4^ with the assumption that non-plastic fibers are readily biodegradable or non-dangerous for the marine biota^56^. Athey and Erdle also point out that, although natural fibers can degrade faster than synthetic polymers, these fibers can persist in the marine environment for decades in relation to the nature of the fiber and environmental factors^55^. Moreover, the degradation of natural and semisynthetic fibers involves release of toxics adsorbed to the surface into the environment^56^. Industrial textile dyes may also protract the microfibers persistence in the environment^55^. We suggest that more attention has to be paid for the experimental detection of dyes in natural fibers, since dyes are indicators of anthropogenic processing and potential causes of biological damage. The effect of natural and semisynthetic microfibers on the marine biota is under studied and deserves particular attention, especially from the polar research community.

### Synthetic microparticles

The synthetic microparticles reported in this study were polyester (PES; 3), polypropylene (PP; 1) polypropylene/polyester (1) and cellulose acetate (1) (Fig. 1).

PES and PP were also the main polymers found in seawater next to the sewage treatment plant at MZS^39^. PP has been also found in sediments of the Ross Sea^38^, as well as in seawater, sediment and sea ice in other Antarctic areas (Table 1). In accordance with our data, one the most common polymers in penguins of Antarctic Peninsula and Scotia Sea region was PP^20^ (Table 2). Fish can act as indirect source of microplastic contamination when preyed by other organisms. The main risks are linked not only to the spread of MPs along the food chain that reaches humans, but above all to the release of dangerous xenobiotics adhered to the MPs^57^.

### Origin of the microfiber contamination

Currently 76 scientific research stations are located below 60° S belonging to 29 nations; 31% are permanent stations whereas 69% of the stations are active only during summer.

The wastewater treatment plants are present only in half of research stations (48%). This is the case of the MZS, which is open from mid-October to mid-February. However, conventional wastewater treatment, including tertiary treatment techniques, may remove about 90% of MPs^58^, and this situation may be exacerbated in remote areas where operational difficulties may reduce treatment efficiency^59^. The non-retained MPs can be released in a largely unaltered state, into the nearshore marine environment having passed through facilities. In the same way, textile dyes are not easily removed by conventional wastewater treatment processes due to their intrinsic properties^60^ (i.e. stability and resistance towards light or oxidizing agents). Based on a very recent study on snow fallen in Antarctica, microplastics and anthropogenic fiber contamination can be related to the Antarctic research stations, and originate from the polar clothing and equipment^23^.

## Discussion

This paper provides a snapshot from the past on the AMs ingestion by specimens of emerald rockcod collected 24 years ago, representing the first evidence of this phenomenon in an Antarctic fish.

Moreover, this finding will be useful to compare the past pollution level with the current situation, by analyzing of gastrointestinal tract of *Trematomus bernacchii* caught during the last Antarctic survey (XXXVII expedition – 2021/2022). This comparison will allow to understand the changes in marine litter pollution over time, especially in the Antarctic ecosystem, with its peculiar environmental features and fragility, worthy to be protected and preserved.

This study further stresses the importance of anthropogenic microparticles and textile dyes monitoring in Antarctica and, hopefully, it will help current policy measures on plastic pollution under the Antarctic Treaty to improve surveillance and promote mitigation actions. In the future, a connection network to retrieve older samples in monitoring pollution changes over time is strongly desirable.

## Methods

### Study area and samplings

**Figure 2:**
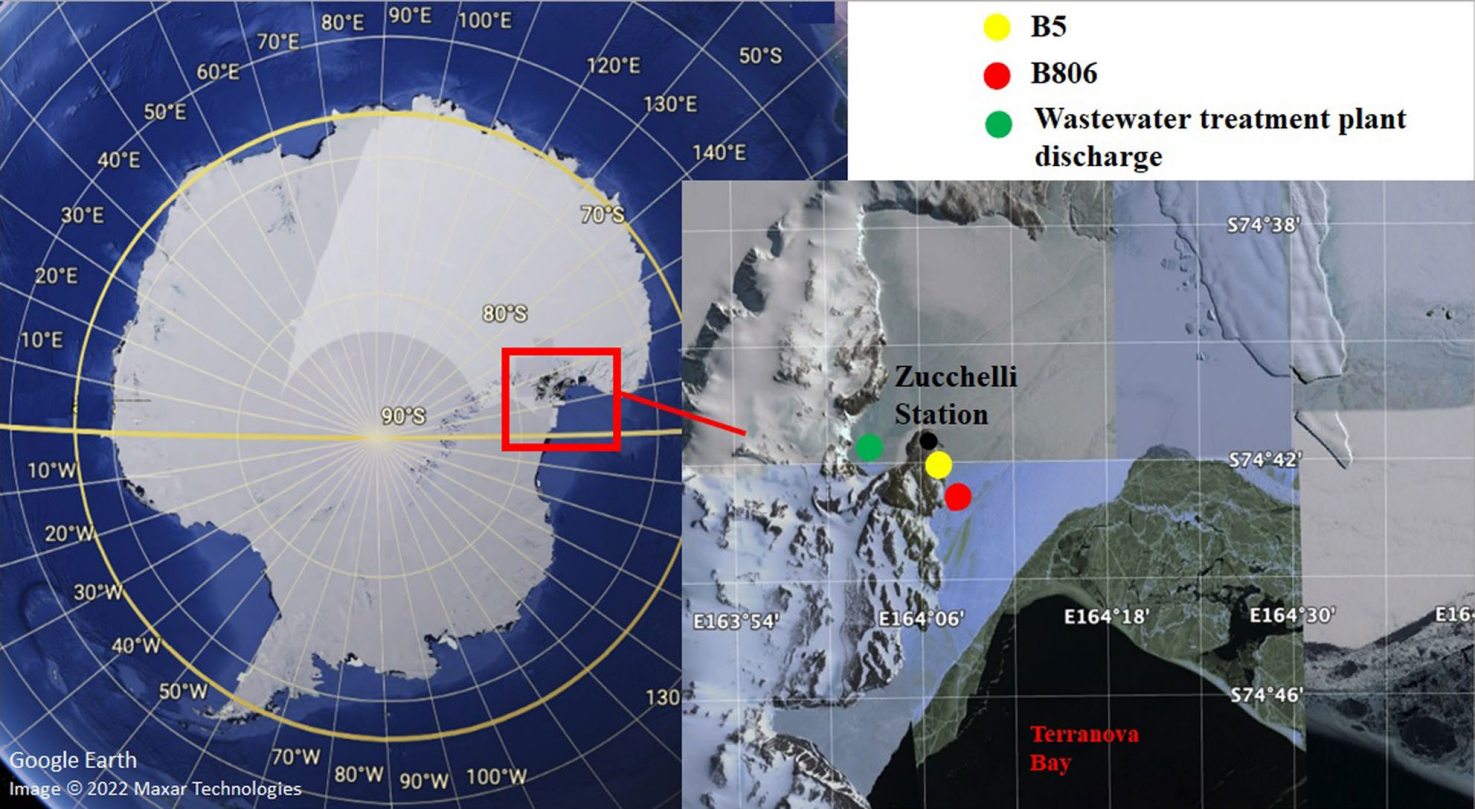
Study area (Terranova Bay, Ross Sea). Map was created using Google Earth (https://earth.google.com/web/@74.6953791,164.0961781,0.4171455a,2467.23579724d,30.00002056y,0h,0t,0r) on which sampling sites and wastewater treatment plant discharge were indicated.

The study area was located in Gerlache Inlet (74° 41′ S; 164° 6′ E), a 7-km wide inlet in the northwest corner of Terra Nova Bay (Ross Sea), a coastal marine area encompassing 29.4 km^2^ between Cape Washington and the Drygalski Ice Tongue (Fig. 2).

This site is an important littoral area for long-term scientific investigations, and it has been proposed as an Antarctic Specially Protected Area (ASPA) by Italy, in 2003. Samplings were carried out on February 1998 in two sites: B5 (74° 41′ 60′′ S, 164° 07′ 00′′ E) and B806 (74° 43′ 025'' S, 164° 08′ 648'' E). B5 and B806 are 3.8 and 7 km from the wastewater outfall, respectively (Fig. 2). The specimens were caught by trammel net, immediately frozen, sent to Italy at the end of the expedition, and finally stored at the Antarctic Environmental Specimen Bank. The samples were kept at -20 °C from the sampling to the analysis, performed in June 2021.

### AMs isolation

Each specimen was slowly thawed at 4 °C overnight, and then GITs, from the oesophagus to the end of the intestine, were removed and stored in a glass Petri dish. Each GIT, was weighted and under a microbiological hood, placed in conical glass flask and treated with 10% K-OH solution, in a ratio of 1: 5 (w/v). The flask was stirred at 50 °C for 48 h. Then each sample was placed in a glass cylinder adding hypersaline 15% NaCl solution to obtain density separation of the two phases. The supernatant was collected in a glass beaker, and filtered through a fiberglass filters (1.6 μm Whatman GF/F, UK) using a vacuum system (Millipore). After filtration procedures, the membranes were placed in sterile Petri glass dishes for subsequent observations under the stereomicroscope (Leica M205C) to isolate the plastic debris^61^. All particles were visually identified, counted, measured, and photographed. All items were classified based on their size (small-microparticle: 0.1–1 mm; large-microparticle: 1–5 mm; meso: 5–25 mm; macro: > 25 mm), shape (pellet, fiber, foam, fragment, sheet and sphere) and colour according to the protocol of the Marine Strategy Framework Directive^62^.

The anthropogenic items found in the GITs were expressed as number of identified AMs per specimens. All items isolated from each fish specimen was assayed for the characterization.

### Preventing contamination

To avoid airborne contamination during laboratory analysis, workspaces and tools were rigorously cleaned from any particle contamination, following succeeding protocol^63^, the samples were processed in a room with restricted access, to prevent any accidental external contamination, and all the operations were performed under a microbiological hood. All materials used for the dissection, the extraction and analysis steps were thoroughly cleaned with ethanol and filtered deionized water. Glassware was used and all instruments and equipment (including tweezers and scissors) were rinsed thoroughly with ultrapure water. Additionally, operators wore cotton coats. The beakers were covered with aluminium foil between each step to prevent airborne contamination. Procedural blanks were also run concurrently to avoid fibers overestimation, moist filters in Petri dishes were put under the microbiological hood and exposed to the laboratory air near the stereomicroscope^64^. Procedural blanks without tissue were also run concurrently with samples. One procedural blank every two samples was performed. The procedural blank samples were free of any AM contamination.

### Analysis of anthropogenic microparticles by Raman micro-spectroscopy

All particles isolated from the GITs were assessed with by Raman micro-spectroscopy. A preliminary analysis of the Raman results was aimed at identifying and grouping the similar spectra. Subsequently, the molecular identification of the component of the AMs was accomplished by comparing the Raman spectra with those available in libraries.

Micro-Raman spectroscopy was used to identify the polymer compositions of the AMs. Fibers showing branching or changed thickness along their length were rejected, both features being evidence for a biological origin (root or plant fibers).

Raman spectra of micro fibers and particles were taken in a backscattering geometry on a HR Evolution micro confocal Raman system (Horiba Scientific) using a green-light-emitting diode laser (λ = 532 nm), a × 100 (NA = 0,90) or a × 50 objective (NA = 0.45), an 1800 g/mm grating and a 77 K—cooled charged couple device detector. To avoid sample photodegradation, the laser power was maintained below 5 mW using a lower percent transmission filter. Spectra were collected in the range of 200–4000 cm^-1^, while the duration of laser exposure and the number of spectra accumulations were varied in the range 5–20 s and 2–20, respectively, depending on the specific dye in the MP. Analysis was performed at different points on the same MP to confirm identification. To remove background interference due to fluorescence, fluorescent samples data were corrected by the Flat correction routine of the LabSpec6. Furthermore, besides FLAT correction, baseline correction was applied prior to the spectral analysis. The spectra were identified by comparing them to those of standard materials catalogued in the spectral databases of the Bio-Rad KnowItAll Spectral Library and the Spectral Library of Plastic Particles (SLoPP and SLoPP-E). A Hit Quality Index (HQI) value of 80% or greater has been accepted as true math.

### Statement of fish specimen permission

The authors declare that they have obtained permission for the use of Antarctic fish samples for research purposes from the Italian National Antarctic Museum (MNA) and from the Bank of Antarctic Environmental Samples (BCAA) of Genoa.

## Data Availability

The datasets used and/or analysed during the current study available from the corresponding author on reasonable request.
